# B cell development is critically dependent on NFATc1 activity

**DOI:** 10.1038/s41423-018-0052-9

**Published:** 2018-06-15

**Authors:** Sabrina Giampaolo, Gabriela Wójcik, Stefan Klein-Hessling, Edgar Serfling, Amiya K. Patra

**Affiliations:** 1 Department of Molecular Pathology, Institute of Pathology, University of Würzburg, Josef Schneider-Str. 2, 97080 Würzburg, Germany; 2 Institute of Translational and Stratified Medicine, Faculty of Medicine and Dentistry, University of Plymouth, Plymouth, PL6 8BU UK; 3 Comprehensive Cancer Center Mainfranken, University of Würzburg, Josef Schneider-Str. 6, 97080 Würzburg, Germany

**Keywords:** Differentiation, EBF1, NFATc1, Pro-B, Pre-B

## Abstract

B cell development in bone marrow is a precisely regulated complex process. Through successive stages of differentiation, which are regulated by a multitude of signaling pathways and an array of lineage-specific transcription factors, the common lymphoid progenitors ultimately give rise to mature B cells. Similar to early thymocyte development in the thymus, early B cell development in bone marrow is critically dependent on IL-7 signaling. During this IL-7-dependent stage of differentiation, several transcription factors, such as E2A, EBF1, and Pax5, among others, play indispensable roles in B lineage specification and maintenance. Although recent studies have implicated several other transcription factors in B cell development, the role of NFATc1 in early B cell developmental stages is not known. Here, using multiple gene-manipulated mouse models and applying various experimental methods, we show that NFATc1 activity is vital for early B cell differentiation. Lack of NFATc1 activity in pro-B cells suppresses EBF1 expression, impairs immunoglobulin gene rearrangement, and thereby preBCR formation, resulting in defective B cell development. Overall, deficiency in NFATc1 activity arrested the pro-B cell transition to the pre-B cell stage, leading to severe B cell lymphopenia. Our findings suggest that, along with other transcription factors, NFATc1 is a critical component of the signaling mechanism that facilitates early B cell differentiation.

## Introduction

B cell development in bone marrow (BM) follows a series of differentiation stages through which hematopoietic stem cell (HSC)-derived common lymphoid progenitor (CLP) cells give rise to mature B lymphocytes. Similar to early T cell development in the thymus, early B cell development in BM is also critically dependent on interleukin-7 (IL-7) signaling.^1–5^ Upon IL-7 signaling, CLP-derived B220^+^CD43^+^ pro-B cells differentiate into B220^+^CD43^−^ pre-B cells, which subsequently differentiate into B220^+^IgM^+^IgD^−^ immature B cells to finally give rise to B220^+^IgM^+^IgD^+^ mature B cells. Pro-B cells can also be characterized as B220^+^IL-7R^+^ because they respond to IL-7 signals or B220^+^CD19^+^IgM^−^c-Kit^+^ cells because they express stem cell factor receptor (c-Kit). Similarly, pre-B cells are otherwise characterized as B220^+^IL-7R^−^ because these cells are independent of IL-7 signaling or B220^+^CD19^+^IgM^−^CD25^+^ because they express the IL-2 receptor alpha chain (CD25). During the pro- to pre-B cell transition, developing B cells rearrange their immunoglobulin (Ig) heavy and light chains, which are essential for the formation of the pre-B cell receptor (pBCR). PreBCR signaling imparts independence from IL-7 signaling and is critical for further differentiation to later developmental stages.^6,7^

Apart from IL-7 signaling, a host of lineage-specific transcription factors (TF) have been implicated in the regulation of early B cell differentiation in BM. Prominent among these TFs are the ets-family TF PU.1, Ikaros, zinc finger TF Bcl11a, helix-loop-helix TF E2A, EBF1 (early B cell factor 1), and paired box TF Pax5.^8,9^ Both E2A and EBF1 have been reported to regulate B lineage specification and commitment, and Pax5 activity is vital for B lineage maintenance.^10–15^ Genetic ablation of these TFs has revealed serious abnormalities in B cell development, leading to severe B cell lymphopenia.^9^

EBF1 activity has been suggested to influence several aspects of early B cell differentiation.^16,17^ EBF1 activity has been shown to regulate *Pax5*, *Pou2af1* (BOB-1/OBF1), and *Foxo1* activity during B cell development.^15,18–21^ The master regulatory function of EBF1 in B lineage specification and commitment was evident from the analysis of *Ebf1*^−/−^ mice, in which development was blocked at the pro-B cell stage.^12^ EBF1 in association with E2A, Runx1, and other TFs regulates a number of B lineage-specifying genes that are involved in early B cell survival, proliferation, and Ig gene locus accessibility and rearrangement.^16,22,23^ Although the functional aspects of EBF1 in B cell development have been extensively studied, the mechanism that induces *Ebf1* expression is less well understood. IL-7 signaling has been shown to regulate *Ebf1* expression in pro-B cells.^24^ Additionally, E2A and FOXO1 appear to regulate *Ebf1* expression.^25–27^ However, a clear picture of the regulation of *Ebf1* during early B cell development has yet to emerge.

Nuclear factor of activated T cell (NFAT) family TFs play important roles in the development and function of many hematopoietic cell lineages.^28–30^ Among five family members (NFATc1, NFATc2, NFATc3, NFATc4, and NFAT5), NFATc1, NFATc2, and NFATc3 are predominantly active in lymphocytes, where they regulate processes as diverse as cell survival, proliferation, cytokine gene expression, various T helper cell differentiation, apoptosis, and immune effector function.^31,32^ In T or B cells, cell surface T cell receptor (TCR) or BCR ligation induces increased calcium (Ca^2+^) flux in the cytoplasm, which requires phospholipase Cγ2 (PLCγ2) activity. Increased Ca^2+^ subsequently activates the serine/threonine phosphatase calcineurin, which by dephosphorylating multiple serine residues in cytoplasmic NFAT proteins facilitates their activation and nuclear translocation. We have recently described an alternative mechanism of NFAT activation in pTCR-negative thymocytes, wherein IL-7-JAK3 activity activates NFATc1 in a tyrosine phosphorylation-dependent manner.^33^ NFAT activity in pTCR-negative thymocytes is not only critical for their survival and differentiation into T cells but is also indispensable in preventing the pathogenesis of T acute lymphoblastic leukemia.^34^

Furthermore, we and others have previously reported the involvement of NFAT proteins in B cell development and functions.^35–38^ Although NFAT proteins regulate multiple events in B cell function, their role in early stages of B cell differentiation in BM has not yet been investigated. Here we show that NFAT is active in pBCR-negative B cells and that NFATc1 plays an indispensable and non-redundant role very early during B cell differentiation. The serious defects in early B cell differentiation in mice lacking NFATc1 activity in the hematopoietic system led to severe B cell lymphopenia, which further underscores the critical necessityof NFATc1 in facilitating B cell development.

## Materials and Methods

### Mice

C57BL/6 wild-type (WT), *Rag1*^−/−^, *Nfatc2*^−/−^, *Nfatc3*^−/−^, *Nfatc2*^−/−^*Nfatc3*^−/−^, *Nfatc1-eGfp-Bac* tg, *Vav*-Cre*Nfatc1*^fl/+^, *Vav*-Cre*Nfatc1*^fl/fl^, *Il7*^−/−^, *Il7r*^−/−^, *Vav*-Cre*Nfatc1P2*^fl/fl^, *Vav*-Cre*Nfatc1*α*A*^fl/+^, and *Vav*-Cre*Nfatc1*α*A*^fl/fl^ mice, all in the C57BL/6 background and of 3–8 weeks age unless mentioned otherwise, were used in the study. All animals were housed in the University of Würzburg central animal facility (ZEMM) following standard animal care procedures. All animals had ad libitum access to food and water. In each experiment, age-matched mice were used without any gender bias, and animals were handled humanely throughout the study.

### Flow cytometry and cell sorting

All antibodies (Abs) used in flow cytometry and for isolation of B220^+^CD43^+^ or B220^+^IL-7R^+^ pro-B and B220^+^CD43^−^ or B220^+^IL-7R^−^ pre-B cells from BM were purchased either from BD Pharmingen or eBioscience. Anti-B220 (RA3^−^6BU), anti-CD43 (S7), anti-CD25 (PC61), anti-CD127 (B12-1), anti-CD3 (145-2C11), anti-IgM (R6-60.2), anti-IgD (11-26c), anti-CD21 (7G6), anti-CD23 (B3B4), anti-CD5 (53–7.3), anti-CD19 (1D3), anti-CD117 (2B8), anti-BP1 (6C3), anti-CD24 (M1/69), and isotype-matched control Abs either directly conjugated to fluorochromes or to biotin were used throughout the study. Biotinylated Abs were revealed with secondary streptavidin-conjugated allophycocyanin or phycoerythrin-Cy5 (PE-Cy5) Abs. Flow cytometry and data analysis were performed following standard procedures using the FACSCalibur and FlowJo software.

For BM pro- and pre-B cell sorting, a single-cell suspension of BM cells from both hind limbs was prepared. WT BM cells were either stained with B220 and CD43 or with B220 and IL-7R Abs to sort B220^+^CD43^+^ or B220^+^IL-7R^+^ pro-B and B220^+^CD43^−^ or B220^+^IL-7R^−^ pre B cells. Similarly, B220^+^CD43^+^ pro-B and B220^+^CD43^−^ pre-B cells were sorted from *Rag1*^−/−^ mice BM. Cell sorting was performed using a FACSAria (BD Biosciences) flow cytometer and FACS DIVA software.

### Immunofluorescence staining and immunoblotting

Sorted BM B220^+^CD43^+^ pro-B and B220^+^CD43^−^ pre-B cells from WT or *Rag1*^−/−^ mice or B220^+^IL-7R^+^ pro-B and B220^+^IL-7R^−^pre-B cells from WT mice were immunostained with NFATc1, NFATc2 (both from ImmunoGlobe), or NFATc3 (Santa Cruz) Abs following a previously published protocol.^39^ Counterstaining with 4,6-diamidino-2-phenylindole was performed to confirm nuclear staining. Image acquisition and data analysis were performed using a TCS SP2 Leica confocal microscope and software.

Whole-cell protein extracts from the WT pro- and pre-B cells were prepared as mentioned previously.^40^ Fifty micrograms of total protein was analyzed in an 8% sodium dodecyl sulfate-polyacrylamide gel electrophoresis (SDS-PAGE) and subjected to immunoblotting for detection of calcineurin (Cell Signaling Technology) and PLCγ2 (Santa Cruz) levels. Actin was used as a loading control.

### In vitro kinase assays

*Rag1*^−/−^ pro-B cells (10^7^) were lysed in modified RIPA buffer, and the cell lysates were incubated with 5 μg JAK3 Ab (Santa Cruz Biotech) overnight (o/n) at 4 °C to immunoprecipitate JAK3. Subsequently, 20 μl protein A agarose beads were added to the cell lysate and incubated for 1 h at 4 °C to capture the immunoprecipitated JAK3. The beads were washed one time in 1× tyrosine kinase buffer (60 mM HEPES, 5 mM MgCl_2_, 5 mM MnCl_2_, 3 μM Na_3_VO_4_, and 2.5 μM DTT), and kinase reactions were set up by adding 10 μg of glutathione *S*-transferase (GST)-NFATc1 or GST-STAT5 (signal transducer and activator of transcription factor 5) protein with 20 μM ATP and 10 μCi γATP in radioactive assays at 22 °C for 30 min. For the non-radioactive assays, kinase reactions were performed without addition of γATP to the reaction mixture. Samples were analyzed by 6–8% SDS-PAGE followed by autoradiography for γ-32P incorporation or immunoblotted to detect phospho-tyrosine.

### V-D-J recombination

For analysis of rearranged Ig heavy and light chain genes, cDNA was synthesized from sorted B220^+^CD43^+^ pro-B cells from WT and *Vav*-Cre*Nfatc1*^fl/fl^ mice using the Miltenyi Biotec cDNA Synthesis Kit and protocol. Semiquantitative reverse transcriptase–polymerase chain reaction (RT-PCR) was performed to measure the expression of various rearranged heavy and light chain genes as well as germ line transcripts, using previously described primer pairs.^41^

### Electrophoretic mobility shift assay (EMSA)

Nuclear extracts (NEs) prepared from freshly isolated BM B220^+^ B cells from *Rag1*^−/−^ mice were analyzed in EMSA for DNA binding of NFATc1 at the *Ebf1* promoter. The following oligonucleotides were used as EMSA probes: *Ebf1* I: 5′-ATCTACACGCACGGAAAGGAAAGAAACATCTTTGGTT-3′, *Ebf1* II: 5′-TGAGAGAGGAGGAGGAAAAAAGAGAGAGAAAAAAACT-3′, and *Ebf1* III: 5′-CTTGCCTGGTTGTGGAAAATGACACTTCAAGT-3′, representing conserved NFAT sites I, II, and III at the *Ebf1* promoter. An oligonucleotide containing the distal NFAT site at the murine *Il2* promoter (*Il2Pu-b*_*d*_) was used as a positive control: (–292) 5′-CCAAAGAGGAAAATTTGTTTCATACAGAAGGC-3′ (–261). Five micrograms of nuclear proteins was incubated with 1 μg poly*-dI*.*dC* and 1 μl γ-32P-labeled *Il2Pu-b*_*d*_ or *Ebf1* probes for 20 min on ice. For competition, a 100-fold molar excess of unlabeled cold probe was used, and in the supershift assays, 3 μg NFATc1, NFATc2, or NFATc3 Abs were used to evaluate the specificity of the NFAT–DNA complexes. DNA-bound NFAT complexes were resolved in 5% non-denaturing gels using 0.5× TBE buffer at 140 V for 2 h. Subsequently, the gels were transferred to Whatman filter papers and vacuum-dried for 2 h. Dried gels were exposed to photographic film o/n at −20 °C, and NFAT–DNA complexes were visualized in the autoradiogram. Free probe: labeled oligos without NE, in each probe set, lane 1: NFAT–DNA complexes, lane 2: competition with cold oligos, lane 3: supershift with NFATc1 Abs, lane 4: supershift with NFATc2 Abs, and lane 5: supershift with NFATc3 Abs.

### Chromatin immunoprecipitation (ChIP)

For each ChIP assay, 5–8 × 10^6^ WT pro-B cells were used following the Miltenyi Biotech ChIP protocol. Eight micrograms of NFATc1 (Santa Cruz Biotech; sc-7294) or GST (A-6; Santa Cruz Biotech) Abs were used for immunoprecipitation o/n at 4 °C. Protein-DNA complexes were precipitated, and DNA fragments bound to NFATc1 or GST were purified. The purified DNA fragments were used in PCR assays to amplify the *Ebf1* promoter region bound to NFATc1 or GST. Primer sequences used to detect the *Ebf1* promoter region are available in Supplementary Table 1.

### Luciferase reporter assays

Murine *Ebf1a* or *Ebf1b* luciferase reporter constructs (100 ng)^42^ were co-transfected independently along with 1000 ng control vectors, expression vectors for a constitutively active STAT5 (STAT5ca) or NFATc1 (Nc1) alone, or with both STAT5 and NFATc1 together into 293 HEK cells using TurboFect Transfection Reagent (Thermo Scientific™, # R0531). At 24 h post-transfection, the cells were left unstimulated or stimulated with 12-*O*-tetradecanoylphorbol-13-acetate plus ionomycin (100 ng/ml each, Calbiochem) in the absence or presence of cyclosporin A (CsA, 100 ng/ml) for 16 h. Subsequently, luciferase activity depicting *Ebf1a* and *Ebf1b* promoter transactivation was measured using a MicroLumat LB 96P (EG&G Berthold) luminometer.

### Photographs

Photographs of the thymus, spleen, and lymph nodes (LNs) from WT, *Vav*-Cre*Nfatc1*^fl/+^, and *Vav*-Cre*Nfatc1*^fl/fl^ mice or the spleen and LNs from WT and *Vav*-Cre*Nfatc1P2*^fl/fl^ mice were obtained using a Nikon Coolpix 4500 digital camera. Image processing was performed using the Adobe Photoshop software.

### Semiquantitative RT-PCR

Sorted B220^+^CD43^+^ pro- and B220^+^CD43^−^ pre-B cells from WT mice, pro-B cells from WT, *Vav*-Cre*Nfatc1*^fl/+^ and *Vav*-Cre*Nfatc1*^fl/fl^ mice, sorted WT double-negative 3 (DN3) thymocytes, and *Vav*-Cre*Nfatc1P2*^fl/fl^ mouse thymocytes and pro-B cells were used to synthesize cDNA using the Miltenyi Biotec cDNA Synthesis Kit and protocol. Semiquantitative RT-PCR was performed to estimate the expression levels of the indicated genes. Primer sequences are available in the supplementary information online.

### Statistics

Data are presented as the mean ± s.d. Statistical significance was assessed using Student’s *t* test for comparison between two groups and analysis of variance for differences among groups.

## Results

### NFAT activation in pro-B cells

**Fig. 1:**
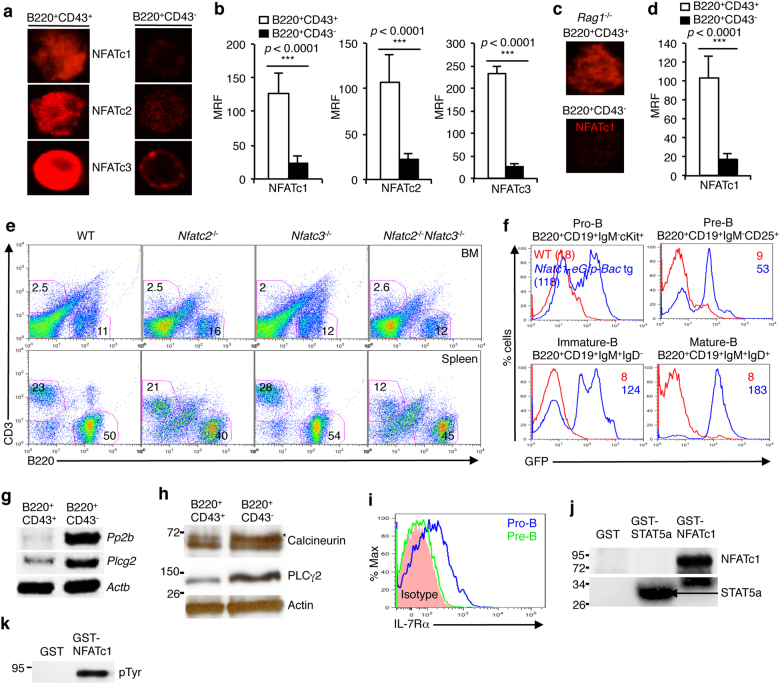
NFAT Expression in pro-B cells. **a** Immunofluorescence revealing the expression of NFATc1, NFATc2, and NFATc3 in WT B220^+^CD43^+^ pro-B and B220^+^CD43^−^ pre-B cells. **b** Quantification of the nuclear NFATc1 (pro-B; *n* = 32 and pre-B; *n* = 81), NFATc2 (pro-B; *n* = 38 and pre-B; *n* = 64), and NFATc3 (pro-B; *n* = 13 and pre-B; *n* = 32) levels in WT  pro- and  pre-B cells. **c** NFATc1 expression levels in *Rag1*^−/−^ pro- and pre-B cells. **d** Quantification of the nuclear NFATc1 (pro-B; *n* = 14 and pre-B; *n* = 28) levels in *Rag1*^−/−^ pro- and pre-B cells. **e** Flow cytometry profiles showing the distribution of CD3^+^ T cells and B220^+^ B cells in the BM and spleen of *Nfatc2*^−/−^, *Nfatc3*^−/−^, and *Nfatc2*^−/−^*Nfatc3*^−/−^ mice compared with WT mice. **f** GFP levels in BM B220^+^CD19^+^IgM^−^c-Kit^+^ pro-B, B220^+^CD19^+^IgM^−^CD25^+^ pre-B, B220^−^CD19^+^IgM^+^IgD^−^ immature B, and B220^+^CD19^+^IgM^+^IgD^+^ mature B cells from *Nfatc1-eGfp-Bac* tg reporter mice compared with WT mice. **g** Levels of *calcineurin* and *Plcg2* mRNA expression in sorted WT B220^+^CD43^+^ pro-B and B220^+^CD43^−^ pre-B cells, as revealed by RT-PCR. **h** Immunoblot analysis of the protein levels of calcineurin and PLCg2 in sorted WT B220^+^CD43^+^ pro-B and B220^+^CD43^−^ pre-B cells. **i** IL-7R expression in WT pro- and pre-B cells. **j** In vitro kinase assays revealing JAK3-mediated phosphorylation of NFATc1 and STAT5. **k** Kinase assay followed by immunoblotting revealing JAK3-mediated tyrosine phosphorylation in NFATc1. Data are representative of three independent experiments (*n* = 3–7 per group) and are shown as the mean ± s.d., unpaired *t* test (**b**, **d**)

To investigate whether TFs of the NFAT family play any role in B cell development in BM, we analyzed WT pro- and pre-B cells for the presence of NFAT proteins. Immunofluorescence analysis of WT B220^+^CD43^+^IgM^−^ pro-B cells revealed many fold higher nuclear NFAT levels compared with B220^+^CD43^−^IgM^+^ pre-B cells (Fig. 1a, b). Confirming this finding, analysis of pro-B (B220^+^IL-7R^+^) and pre-B (B220^+^IL-7R^−^) cells isolated based on IL-7R expression revealed a similar NFAT distribution pattern (Supplementary Figure S1a and b). Irrespective of the markers used to identify pro- and pre-B cells, NFAT activation in pBCR-negative pro-B cells was higher for NFATc1, NFATc2, and NFATc3 compared with that in pBCR-positive pre-B cells (Fig. 1a, b and Supplementary Figure S1a and b). This observation was further consolidated by analyses of *Rag1*^−/−^ pro-B cells. Similar to WT pro-B cells, *Rag1*^−/−^ pro-B cells also exhibited high levels of nuclear NFATc1 (Fig. 1c, d), suggesting that NFAT proteins most likely play a role at the preBCR-negative stages of B cell development in BM.

Owing to the high levels of NFATc1, NFATc2, and NFATc3 in pro-B cells, we next investigated which of the three NFAT family members is critical for early B cell development. Analysis of the B cell population in the BM of *Nfatc2*^−/−^ or *Nfatc3*^−/−^ mice revealed comparable proportions of B220^+^ cells as in WT mice (Fig. 1e). Even the combined loss of both NFATc2 and NFATc3 (*Nfatc2*^−/−^*Nfatc3*^−/−^) did not alter B220^+^ population in BM (Fig. 1e), suggesting that NFATc2 and NFATc3 activity was most likely dispensable and that NFATc1 was the key NFAT protein regulating early B cell differentiation. This observation was supported by an earlier report that did not reveal any abnormality in early B cell development in *Nfatc2*^−/−^, *Nfatc3*^−/−^, or the double-mutant mice, although mature splenic B cell function was affected.^36^ NFATc1 expression in early B cell developmental stages was further confirmed, as B220^+^CD19^+^IgM^−^c-Kit^+^ pro-B cells from *Nfatc1*-*eGfp*-*Bac* transgenic (tg) reporter mice^34,37,43^ showed high GFP levels compared with those from WT mice (Fig. 1f and Supplementary Figure S1c). In fact, varying levels of GFP representing NFATc1 expression in pro-B, B220^+^CD19^+^IgM^−^CD25^+^ pre-B, B220^+^CD19^+^IgM^+^IgD^−^ immature B, and B220^+^CD19^+^IgM^+^IgD^+^ mature B cells in BM (Fig. 1f and Supplementary Figure S1c) and in splenic B cells (Supplementary Figure S1d) suggest that NFATc1 activity plays an essential role in B cell development and function.

To gain insight into how NFAT is activated in pro-B cells, we analyzed the expression of components of the classical NFAT activation pathway, i.e., the PLCγ2–calcium–calcineurin pathway. RT-PCR as well as immunoblot analysis showed that, compared with pre-B cells, pro-B cells expressed very low levels of *Plcg2* and *calcineurin* (*Pp2b*) (Fig. 1g, h). Considering the high levels of NFAT (Fig. 1a–d and Supplementary Figure S1a and b) and insignificant expression of *Plcg2* and *calcineurin*, it was apparent that NFAT activation was calcineurin independent in pro-B cells. This finding was supported by observations from previous reports showing normal early B cell development in the BM of calcineurin-deficient mice.^44,45^

To understand the mechanism of NFAT activation, we investigated whether IL-7-JAK3 signaling also activates NFATc1 in pro-B cells as we have observed in thymocytes.^33^ Pro-B cells depend on IL-7 signaling for survival and differentiation.^46,47^ Accordingly, we detected high levels of IL-7R expression on pro-B compared with pre-B cells (Fig. 1i). To characterize the involvement of JAK3, we performed kinase assays with JAK3 immunoprecipitated from pro-B cells. In kinase assays, JAK3 phosphorylated NFATc1 as well as STAT5, indicating that, similar to observations in thymocytes, JAK3 plays the role of NFAT kinase, resulting in NFAT activation and nuclear translocation in pro-B cells (Fig. 1j). JAK3-mediated tyrosine phosphorylation of NFATc1 was confirmed by immunoblotting the kinase assay products with pTyr Abs (Fig. 1k). These observations suggest that NFAT activation in pro-B cells is dependent on IL-7-JAK3 signaling.

### Impaired B cell development in the absence of NFATc1

**Fig. 2:**
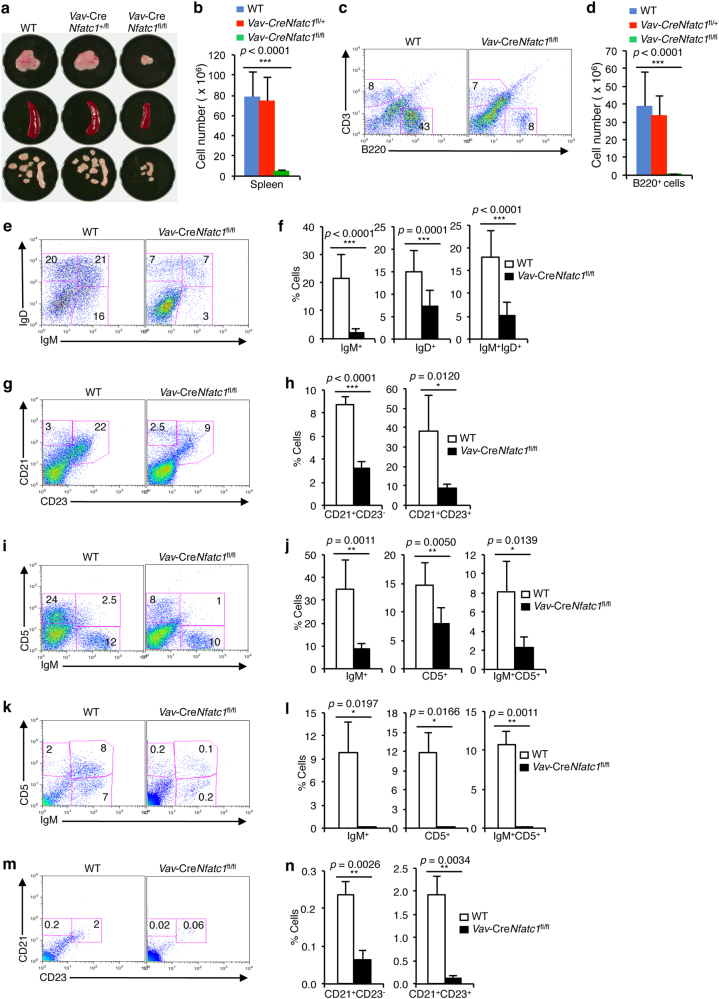
Impaired B cell development in *Vav*-Cre*Nfatc1*^fl/fl^ mice. **a** Photographs of the thymus, spleen, and LNs from *Vav*-Cre*Nfatc1*^fl/fl^ mice compared with littermate *Vav*-Cre*Nfatc1*^fl/+^ and WT mice. **b** Total splenocyte numbers in WT (*n* = 8), *Vav*-Cre*Nfatc1*^fl/+^ (*n* = 8), and *Vav*-Cre*Nfatc1*^fl/fl^ (*n* = 9) mice. **c** Flow cytometry showing the distribution of CD3^+^ T and B220^+^ B cells in the spleen from *Vav*-Cre*Nfatc1*^fl/fl^ mice compared with WT controls. **d** Absolute numbers of B220^+^ B cells in the spleen from *Vav*-Cre*Nfatc1*^fl/fl^ (*n* = 9) mice compared with littermate control (*n* = 8 for both *Vav*-Cre*Nfatc1*^fl/+^ and WT) mice. **e**, **f** Distribution of IgM^+^ and IgD^+^ B cells in the spleen (**e**) and quantification of the % cells (**f**) from WT and *Vav*-Cre*Nfatc1*^fl/fl^ mice. **g**, **h** Follicular and marginal zone B cell distribution in the spleen (**g**) and quantification of the percentage of cells (**h**) from *Vav*-Cre*Nfatc1*^fl/fl^ mice compared with littermate control mice as revealed by CD21 and CD23 staining. **i**, **j** Distribution of splenocytes according to CD5 and IgM expression (**i**) and quantification of the percentage of cells (**j**) in WT and *Vav*-Cre*Nfatc1*^fl/fl^ mice. **k**, **l** Distribution of CD5^+^ and IgM^+^ cells (**k**) and quantification of the percentage of cells (**l**) in the peritoneum of *Vav*-Cre*Nfatc1*^fl/fl^ mice compared with WT controls. **m**, **n** Distribution of follicular and marginal zone B cells (**m**) and quantification of the percentage of cells (**n**) in the peritoneum of *Vav*-Cre*Nfatc1*^fl/fl^ mice compared with WT controls. Numbers within each dot plot represent the percentage of the respective population. Data represent one of the three independent experiments (*n* = 3 per group) and are shown as the mean ± s.d., one-way ANOVA (**b**, **d**), and unpaired *t*-test (**f**, **h**, **j**, **l**, **n**)

NFATc1-deficient mice are embryonic lethal.^48^ To circumvent this problem and investigate the influence of NFATc1 on early B cell developmental stages, we analyzed *Vav*-Cre*Nfatc1*^fl/fl^ mice, in which NFATc1 activity was ablated in the hematopoietic system. Analysis of *Vav*-Cre*Nfatc1*^fl/fl^ mice showed a consistent and strong reduction in the size of primary lymphoid organs, such as the thymus, LNs, and spleen (Fig. 2a). A reduction of the thymus size in *Vav*-Cre*Nfatc1*^fl/fl^ mice due to developmental defects in thymocyte differentiation has been reported.^33^ The small LNs and spleen in *Vav*-Cre*Nfatc1*^fl/fl^ mice suggest possible defects in B cell development, as B cells constitute a major cell population in these organs. Accordingly, spleen cellularity was drastically reduced in *Vav*-Cre*Nfatc1*^fl/fl^ mice compared with their littermate controls (Fig. 2b). Analysis of T and B cell distributions in the spleen revealed a strongly reduced B220^+^ B cell population in *Vav*-Cre*Nfatc1*^fl/fl^ mice (Fig. 2c, d). Further assessment of IgM and IgD-expressing B cells in the spleen confirmed that the loss of NFATc1 activity exerted a strong negative influence on B cell development (Fig. 2e, f).

In the absence of NFATc1 activity, very few CD21^+^CD23^+^ follicular and CD21^+^CD23^−^ marginal zone B cells, as well as B220^+^CD5^+^ B-1 B cells, developed in *Vav*-Cre*Nfatc1*^fl/fl^ mice (Fig. 2g–n). Additionally, IgM^+^ B cells in the peritoneum were markedly reduced in *Vav*-Cre*Nfatc1*^fl/fl^mice compared with WT control mice (Fig. 2k, l). This strong phenotype in the absence of NFATc1 activity further confirmed that NFATc1 is the key NFAT protein essential for B cell development, and NFATc2 or NFATc3 cannot compensate for its loss. The reduced B220^+^CD5^+^ population in *Vav*-Cre*Nfatc1*^fl/fl^ mice is in agreement with previous reports concerning the requirement for NFATc1 for the development of these cells.^35^ Taken together, these abnormalities in B cell populations in the peripheral lymphoid organs of *Vav*-Cre*Nfatc1*^fl/fl^ mice indicate a defect in B lymphopoiesis in the BM.

### B cell development in BM is critically dependent on NFATc1 activity

**Fig. 3:**
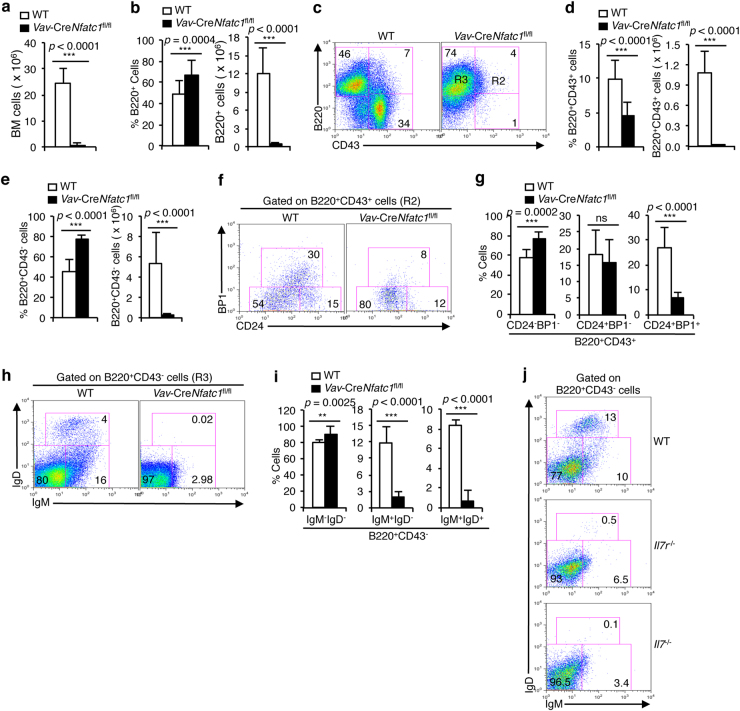
Lack of NFATc1 activity arrests pro-B cell differentiation to pre-B cells. **a** Cellularity in the BM of WT and *Vav*-Cre*Nfatc1*^fl/fl^ mice. **b** B220^+^ B cell distribution both as a percentage of the population and as the absolute numbers in the BM of WT and *Vav*-Cre*Nfatc1*^fl/fl^ mice. **c** Flow cytometry profiles showing the distribution of BM cells based on B220 and CD43 expression in WT and *Vav*-Cre*Nfatc1*^fl/fl^ mice. **d** Quantification of the percentage of distribution and absolute numbers of B220^+^CD43^+^ pro-B cells in the BM of WT and *Vav*-Cre*Nfatc1*^fl/fl^ mice. **e** Quantification of the percentage of distribution and absolute numbers of B220^+^CD43^−^ pre-B cells in the BM of WT and *Vav*-Cre*Nfatc1*^fl/fl^ mice. **f**, **g** B220^+^CD43^+^CD24^−^BP1^−^ pre-pro-B, B220^+^CD43^+^CD24^+^BP1^−^ pro-B, and B220^+^CD43^+^CD24^+^BP1^+^ large pre-B cell distributions in the BM (**f**) and the quantification of their percentage of distribution (**g**) from *Vav*-Cre*Nfatc1*^fl/fl^ mice compared with WT controls. **h**, **i** B220^+^CD43^−^IgM^−^IgD^−^ small pre-B, B220^+^CD43^−^IgM^+^IgD^−^ immature B, and B220^+^CD43^−^IgM^+^IgD^+^ mature B cell distributions in BM (**h**) and the quantification of their percentage of distribution (**i**) from WT and *Vav*-Cre*Nfatc1*^fl/fl^ mice. **j** Distribution of B cells gated on the B220^+^CD43^−^ population in BM from *Il7*^−/−^ and *Il7r*^−/−^ mice based on IgM and IgD expression compared with littermate WT mice. Numbers inside each dot plot represent the percentage of the respective population. Data represent one of three independent experiments (*n* = 3 per group) and are shown as the mean ± s.d., unpaired *t* test

To elucidate the precise stage at which B cell development is affected owing to the absence of NFATc1 activity, we analyzed B cell differentiation in the BM of *Vav*-Cre*Nfatc1*^fl/fl^ mice. BM cellularity as well as BM B220^+^ B cell numbers were dramatically reduced in *Vav*-Cre*Nfatc1*^fl/fl^ mice, although an increase in the percentage of distributed B220^+^ cells was observed (Fig. 3a, b). Further analysis revealed a strong reduction in both the percentage of distribution and in the absolute numbers of the BM B220^+^CD43^+^ pro-B cells (Fig. 3c, d), as well as an increase in the percentage but a marked reduction in the absolute numbers of B220^+^CD43^−^ pre-B cells (Fig. 3c, e) in *Vav*-Cre*Nfatc1*^fl/fl^ compared with WT mice. The B220^+^CD43^+^ population can be further subdivided into three subsets based on the surface expression of heat-stable antigen (CD24) and BP1 molecules, i.e., B220^+^CD43^+^CD24^−^BP1^−^ pre-pro-B cells, B220^+^CD43^+^CD24^+^BP1^−^ pro-B cells, and B220^+^CD43^+^CD24^+^BP1^+^ large pre-B cells.^49^ Our analysis revealed that, in *Vav*-Cre*Nfatc1*^fl/fl^ mice, B cell development was arrested at the B220^+^CD43^+^CD24^−^BP1^−^ pre-pro-B cell stage (Fig. 3f, g). Very few cells differentiated to the later stages, resulting in a dramatically reduced B cell population in the BM and peripheral lymphoid organs (Figs. 3b and 2d). Similar to the B220^+^CD43^+^ population, the B220^+^CD43^−^ population can be divided into three subsets based on the expression of IgM and IgD molecules: B220^+^CD43^−^IgM^−^IgD^−^ small pre-B, B220^+^CD43^−^IgM^+^IgD^−^ immature B, and B220^+^CD43^−^IgM^+^IgD^+^ mature B cells.^49^ In *Vav*-Cre*Nfatc1*^fl/fl^ compared with their littermate control mice, there were very few B220^+^CD43^−^IgM^+^IgD^−^ immature and B220^+^CD43^−^IgM^+^IgD^+^ mature B cells, confirming a developmental arrest at the pro-B cell stage due to the absence of NFATc1 activity (Fig. 3h, i).

We have previously shown that the abolition of NFATc1 activity in B cells (*Mb1*-Cre*Nfatc1*^fl/fl^ mice) leads to defects in mature B cell function, but it does not influence the early B cell differentiation stages in BM.^37^ Correlating our observations with that from *Mb1*-Cre*Nfatc1*^fl/fl^ mice, it is apparent that NFATc1 activity at a stage where the immature B cells still have not acquired the pBCR is critical for B cell development. To investigate the basis behind the differentiation block, we assumed that most likely lack of NFATc1 activity is impairing the IL-7 responsive stages of B cell development in *Vav*-Cre*Nfatc1*^fl/fl^ mice. Furthermore, analysis of *Il7r*^−/−^ and *Il7*^−/−^ mice revealed a similar developmental arrest at the early B cell differentiation stage, as observed in *Vav*-Cre*Nfatc1*^fl/fl^ mice (Fig. 3j and Supplementary Figure S2a). These observations suggest that NFATc1 activation is indispensable for B cell development in BM.

### Loss of NFATc1 dysregulates *Ebf1* and the expression of other B lineage-specific genes

**Fig. 4:**
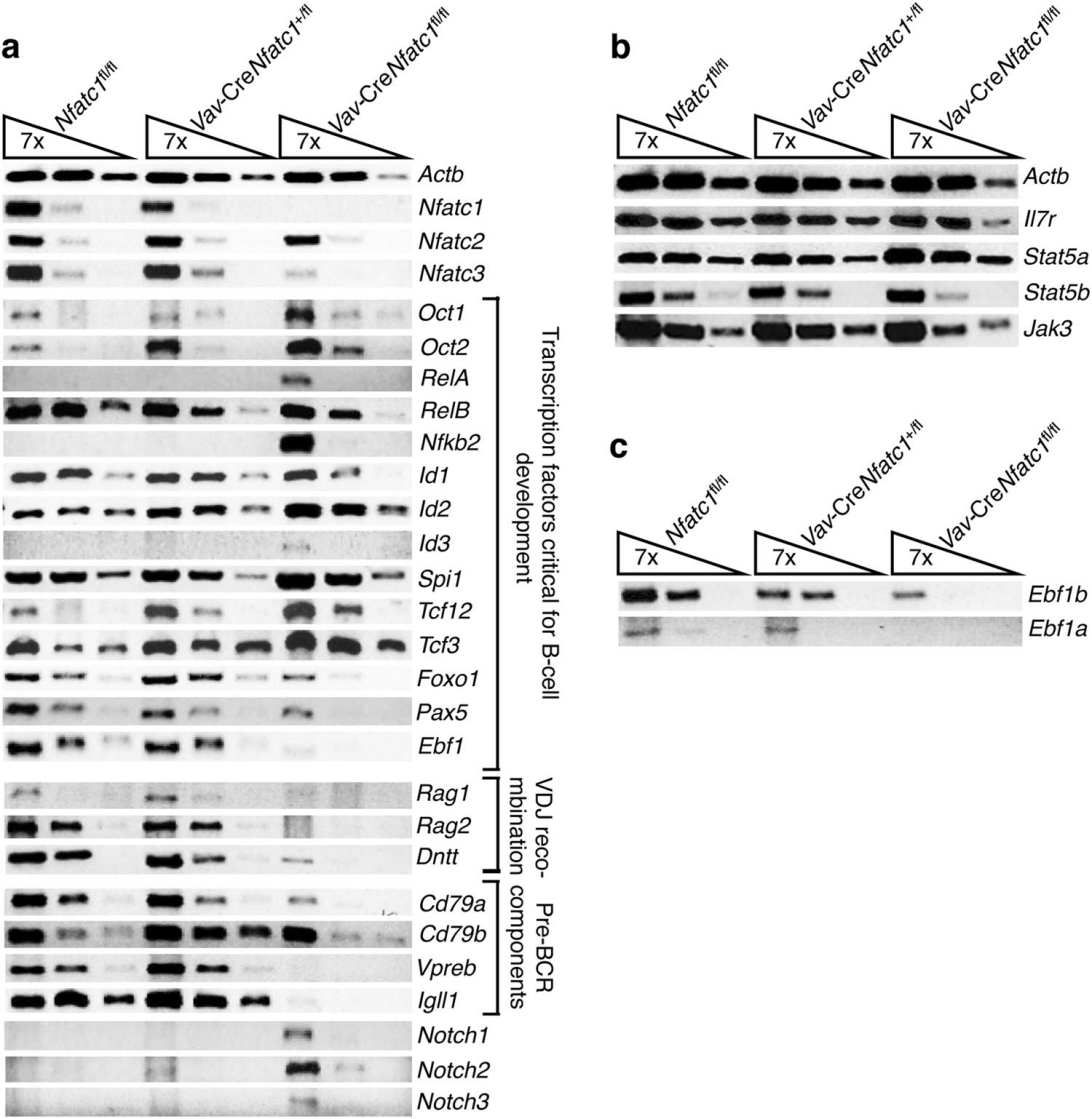
Abnormal B cell development-specific gene expression in *Vav*-Cre*Nfatc1*^fl/fl^ pro-B cells. **a** RT-PCR analysis of gene expression, including transcription factors that regulate B cell development, molecules associated with V-D-J recombination, pBCR components, and other signaling molecules, in *Vav*-Cre*Nfatc1*^fl/fl^ pro-B cells compared with littermate control mice. **b** *Il7r*, *Jak3*, *Stat5a*, and *Stat5b* gene expression in pro-B cells from the indicated mice. **c** Levels of *Ebf1a* and *Ebf1b* isoform expression in *Vav*-Cre*Nfatc1*^fl/fl^ mice compared with littermate control mice. Data are representative of two independent experiments with cDNA prepared from sorted cells collected from pooled BM cells from multiple mice for each genotype in each experiment

To gain insight into the defects in pro-B cell development in the absence of NFATc1 activity, we analyzed the expression of various molecules implicated to regulate B cell development. RT-PCR analysis revealed NFATc1 was specifically and efficiently deleted in pro-B cells of *Vav*-Cre*Nfatc1*^fl/fl^ mice (Fig. 4a). However, normal expression of NFATc2 and reduced expression of NFATc3 were not sufficient to restore the B cell phenotype in *Vav*-Cre*Nfatc1*^fl/fl^ mice, ruling out any compensation by these NFAT family members (Fig. 4a). Interestingly, analysis of molecules that are critically involved in B lineage commitment and specification did not reveal any alterations in the expression of *Spi1* (PU.1), *Tcf3* (E47), *Tcf12* (HEB), *Oct1*, *Oct2*, *Id1*, *Id2*, and *Id3*, ruling out any involvement of NFATc1 in the regulation of these genes (Fig. 4a). In fact, all these genes were slightly upregulated in *Vav*-Cre*Nfatc1*^fl/fl^ pro-B cells. Several NF-κB family members have been shown to regulate B cell development, as evidenced in studies examining gene-deficient mice.^50,51^ However, we observed an elevated level of *Rela* (*P65*), *Relb*, and *Nfkb2* expression in *Vav*-Cre*Nfatc1*^fl/fl^ compared with WT mouse pro-B cells (Fig. 4a). Interestingly, we observed a strong downregulation of *Ebf1* expression as well as reduced expression of *Foxo1* and *Pax5* in *Vav*-Cre*Nfatc1*^fl/fl^ compared with WT mouse pro-B cells (Fig. 4a). Reduced Pax5 expression could be due to a downregulation of EBF1 activity, as it has been reported that *Pax5* expression is regulated by EBF1.^16^ Thus EBF1 seems to be the prime target of NFATc1 during pro-B cell development.

Based on our analysis, the B cell developmental defect in *Vav*-Cre*Nfatc1*^fl/fl^ mice clearly occurs at a similar stage as that in IL-7- or IL-7R-deficient mice (Fig. 3j and Supplementary Figure S2a).^3,4^ To exclude the possibility that the absence of NFATc1 activity impairs IL-7-STAT5 signaling, we investigated the expression of all components of the IL-7 signaling pathway. Normal expression of *Il7ra*, *Jak3*, *Stat5a*, and *Stat5b* genes in *Vav*-Cre*Nfatc1*^fl/fl^ compared with WT mouse pro-B cells (Fig. 4b) excluded the possibility that the absence of STAT5 activity was responsible for the arrested B cell development in *Vav*-Cre*Nfatc1*^fl/fl^ mice. The B cell developmental arrest in IL-7- and IL-7R-deficient mice has been attributed to the absence of EBF1 expression.^24^ The lack of EBF1 expression in *Vav*-Cre*Nfatc1*^fl/fl^ mice (Fig. 4a) resembles to that in IL-7 and IL-7R-deficient mice and suggests that NFATc1 is an important downstream target of IL-7 signaling. 

In addition to the defect in *Ebf1* expression, pro-B cells in *Vav*-Cre*Nfatc1*^fl/fl^ displayed a strong downregulation of the *Rag1*, *Rag2*, and *Tdt* (*Dntt*) genes involved in the recombination and rearrangement of Ig genes. Furthermore, *Vav*-Cre*Nfatc1*^fl/fl^ pro-B cells lacked expression of λ5 (*Igll1*), *VpreB*, Ig-α (*Cd79a*), and Ig-β (*Cd79b*), all of which are components of the pBCR complex. These observations suggest that NFATc1 is a critical player in B cell development, and the absence of NFATc1 can have profound effect on B lymphopoiesis. We also observed similar defects in early-B cell differentiation-specific gene expression in IL-7 signaling-deficient pro-B cells, which was in agreement with previously published reports (Supplementary Figure S2b).

EBF1 expression in pro-B cells is regulated from two distinct promoters, leading to the expression of EBF1α and EBF1β isoforms.^42^ It has been reported that EBF1β is the major isoform expressed in pro-B cells and regulates the expression of EBF1α. In investigations to determine which EBF isoform was affected by the absence of NFATc1, we observed that both *Ebf1a* and *Ebf1b* were strongly downregulated in *Vav*-Cre*Nfatc1*^fl/fl^ mouse pro-B cells (Fig. 4c), suggesting that EBF1 is a target of NFATc1 in pro-B cells.

### NFATc1 regulates EBF1 expression in pro-B cells

**Fig. 5:**
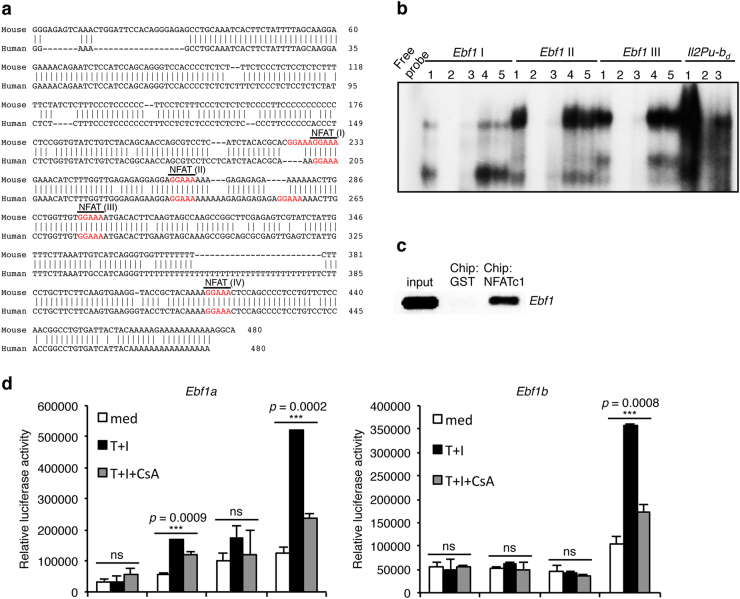
NFATc1 binds the *Ebf1* promoter and regulates its activity. **a** Conserved NFAT-binding consensus sequence (5′-GGAAA-3′) in the *Ebf1b* promoter region 1-kb upstream of the transcriptional start site in mice and humans. **b** Electrophoretic mobility shift assay (EMSA) with nuclear extracts from BM pro-B cells showing NFATc1 binding at the conserved NFAT consensus sites in the *Ebf1* promoter region. *Ebf1* I, *Ebf1* II, and *Ebf1* III indicate the oligos used in EMSA containing the first, second, and third NFAT-binding sites. In each set, lane 1 represents NFAT–DNA complexes; lane 2, competition with 100-fold excess corresponding cold oligos; lane 3, supershift of NFAT–DNA complexes with NFATc1 Abs; lane 4, supershift of NFAT–DNA complexes with NFATc2 Abs; and lane 5, supershift of NFAT–DNA complexes with NFATc3 Abs. In the case of *Il2Pu* box-distal (*Il2Pu-b*_*d*_), lanes 1–3 represent NFAT–DNA complexes, cold oligo competition, and supershift with NFATc1 Abs, respectively. **c** In vivo NFATc1 binding at the *Ebf1* promoter as revealed in the ChIP assays. **d** Transactivation of *Ebf1a* and *Ebf1b* promoters in response to NFATc1 or STAT5 activity alone or both together as revealed by luciferase reporter assays. Data are representative of three independent experiments and are shown as the mean ± s.d., one-way ANOVA (**d**)

To investigate whether NFATc1 transcriptionally regulates EBF1 expression, we searched for the NFAT DNA-binding consensus sequence 5′-GGAAA-3′ approximately 1 kb upstream of the *Ebf1b* transcriptional start site. We detected five NFAT consensus sequences in this upstream sequence, four of which were conserved in mouse and human (Fig. 5a). To verify NFATc1 binding to these conserved sites, we performed EMSAs with NE prepared from WT pro-B cells. As shown in Fig. 5b, NFATc1 binding was detected in sites I, II, and III, with the second and third conserved sites showing a higher affinity in comparison to site I. NFATc1 binding to site IV was weaker than to the other three conserved sites (data not shown). NFATc1 binding to these sites was abolished specifically by an Ab against NFATc1, whereas the complex remained unaltered in the presence of Abs against either NFATc2 or NFATc3 (Fig. 5b). Furthermore, strengthening our observations regarding NFATc1-mediated regulation of *Ebf1* expression, ChIP assays revealed the in vivo NFATc1 binding at the *Ebf1* promoter in WT pro-B cells (Fig. 5c). Supporting these observations,  in reporter assays, we observed minimal transactivation of the *Ebf1b* promoter by NFATc1 (Fig. 5d). However, NFATc1 in combination with STAT5 increased *Ebf1b* promoter activity by several fold, and this effect was reduced when NFATc1 activity was blocked by CsA treatment (Fig. 5d). Interestingly, NFATc1 exerted a similar influence on the *Ebf1a* promoter, suggesting that both *Ebf1a* and *Ebf1b* are transcriptionally regulated by NFATc1 (Fig. 5d), which supports our observations of strongly reduced expression of both isoforms in *Vav*-Cre*Nfatc1*^fl/fl^ pro-B cells (Fig. 4c). These observations suggest that, downstream of IL-7 signals, NFATc1 and STAT5 synergistically regulate *Ebf1* activity and critically influence early B cell differentiation in BM.

### NFATc1 regulates Ig gene rearrangement in pro-B cells

**Fig. 6:**
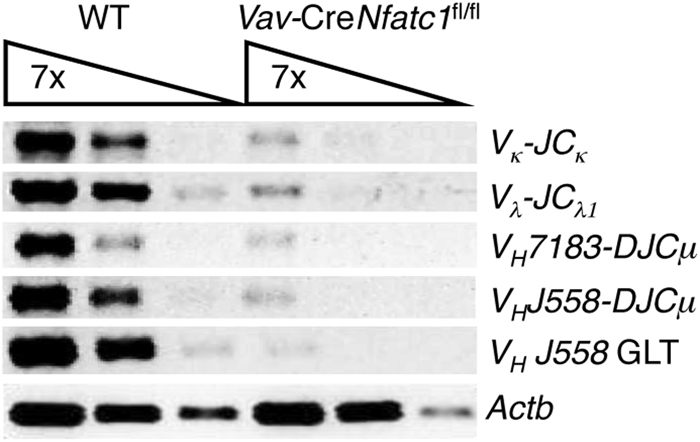
NFATc1 deficiency impairs V-D-J recombination in *Vav*-Cre*Nfatc1*^fl/fl^ pro-B cells. RT**-**PCR analysis showing V-J and V-D-J recombination at the light and heavy chain locus, respectively, in pro-B cells from *Vav*-Cre*Nfatc1*^fl/fl^ mice compared with littermate control mice. Data are representative of two independent experiments with cDNA prepared from sorted cells collected from pooled BM cells from multiple mice for each genotype in each experiment

As B cell development in *Vav*-Cre*Nfatc1*^fl/fl^ mice was arrested at the pro-B cell stage, we analyzed the rearrangement status of the IgH and IgL chain locus. RT-PCR analysis revealed that, compared with WT pro-B cells, transcripts for either κ- or λ-light chains as well as the proximal and distal V_H_ segment containing the IgH chain were negligible in *Vav*-Cre*Nfatc1*^fl/fl^ pro-B cells (Fig. 6). Correspondingly, germline transcripts for distal V_H_ segments were absent in *Vav*-Cre*Nfatc1*^fl/fl^ pro-B cells, indicating a defect in Ig heavy chain locus accessibility (Fig. 6) and raising the possibility of NFATc1 acting as a facilitator of Ig gene locus accessibility and rearrangement. This result further supports a role for NFATc1 downstream of IL-7 signaling in the regulation of B cell developmental events, as IL-7 signaling has been reported to regulate IgH locus accessibility.^52,53^

### A threshold level of NFATc1 activity is essential for B cell differentiation

**Fig. 7:**
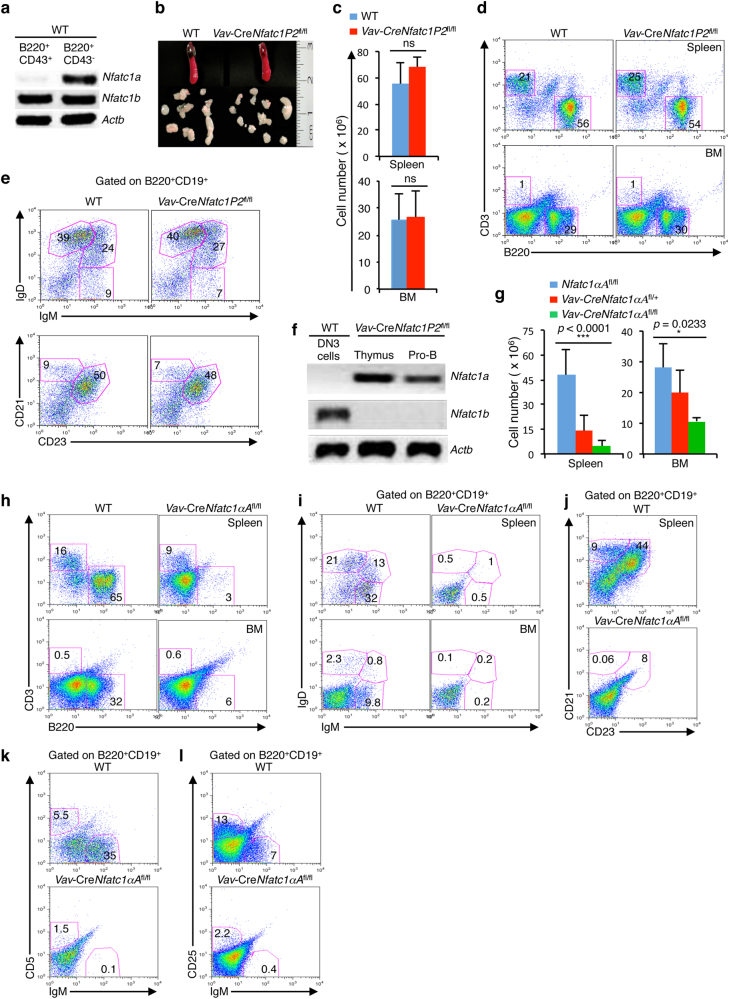
Threshold level of NFATc1 activity is critical for B cell development. **a** Expression levels of *Nfatc1a* and *Nfatc1b* transcripts in B220^+^CD43^+^ pro-B and B220^+^CD43^−^ pre-B cells from WT mice. **b** Photographs of the spleen and LNs from *Vav*-Cre*Nfatc1P2*^fl/fl^ mice compared with littermate control mice. **c** Cellularity in the spleen and BM from the indicated mice (*n* = 3 in each group). **d** CD3^+^ T and B220^+^ B cell distribution in the spleen and BM from *Vav*-Cre*Nfatc1P2*^fl/fl^ mice compared with WT control mice. **e** Splenic distribution of immature and mature (IgM vs IgD staining) and follicular and marginal zone (CD21 vs CD23 staining) B cells in WT and *Vav*-Cre*Nfatc1P2*^fl/fl^ mice as revealed by flow cytometry. **f** Levels of *Nfatc1a* and *Nfatc1b* transcripts in WT DN3 (CD4^−^CD8^−^CD44^−^CD25^+^) thymocytes and thymocytes and sorted pro-B cells from *Vav*-Cre*Nfatc1P2*^fl/fl^ mice. **g** Cellularity in the spleen and BM from *Vav*-Cre*Nfatc1*α*A*^fl/fl^ mice (*n* = 12) compared with littermate *Vav*-Cre*Nfatc1*α*A*^fl/+^ (*n* = 10) and WT mice (*n* = 9). **h** Flow cytometric profiles of the T and B cell distribution in the spleen and BM from *Vav*-Cre*Nfatc1*α*A*^fl/fl^ mice compared with littermate control mice. **i** IgM^+^ and IgD^+^ B cell distribution in the spleen and BM of *Vav*-Cre*Nfatc1*α*A*^fl/fl^ and WT mice gated on the B220^+^CD19^+^ population. **j** Follicular and marginal zone B cell distribution in the spleen from the indicated mice gated on the B220^+^CD19^+^ population. **k** Distribution of CD5^+^ and IgM^+^ cells among the B220^+^CD19^+^ population in the spleen from *Vav*-Cre*Nfatc1*α*A*^fl/fl^ and WT mice. **l** Flow cytometric profiles showing the distribution of B220^+^CD19^+^IgM^−^CD25^+^ pre-B cells and B220^+^CD19^+^IgM^+^CD25^−^ immature B cells in the BM from *Vav*-Cre*Nfatc1*α*A*^fl/fl^ mice compared with littermate control mice. Data represent one of the two (**b**) or three independent experiments (*n* = 3–7 per group) and are shown as the mean ± s.d., paired *t* test (**c**), ns not significant, and one-way ANOVA (**g**)

NFATc1 is expressed from two distinct promoters. NFATc1α isoforms are directed from the distal P1 and NFATc1β isoform expression is directed from the proximal P2 promoter.^54^ We have recently demonstrated distinct developmental stage-specific *Nfatc1* promoter activity during thymocyte differentiation with NFATc1β isoforms expressed in pTCR-negative cells and both NFATc1α and NFATc1β activity in pTCR-positive thymocytes.^34^ To assess whether a similar pattern of NFATc1 promoter activity occurs during B cell development, we evaluated *Nfatc1a* and *Nfatc1b*-specific transcripts in WT pro- and pre-B cells. We observed strong P2 promoter-derived NFATc1β expression in B220^+^CD43^+^ pro-B cells, whereas pBCR-positive B220^+^CD43^−^ pre-B cells showed strong expression of both NFATc1β and P1 promoter-derived NFATc1α (Fig. 7a). Thus NFATc1β isoforms probably play a critical role in the differentiation of pro-B cells in the absence of pBCR signaling, and pBCR-bearing B cells require both NFATc1β and NFATc1α for further differentiation into mature B cells.

To further investigate whether exclusive NFATc1β activity is really critical, and whether B cell differentiation is impaired in the absence of P2 promoter activity, we analyzed *Vav*-Cre*Nfatc1P2*^fl/fl^ mice.^34^ However, preliminary analysis of the LNs, spleen, and BM in these mice did not reveal any perturbations in size or cellularity, suggesting that B cell differentiation was normal in the absence of P2-derived NFATc1β (Fig. 7b, c). Further characterization revealed a normal distribution of B220^+^ B cells in the BM and spleen of these mice (Fig. 7d and Supplementary Figure S3a). Additionally, the unaffected distribution of B220^+^IgM^+^ and B220^+^IgD^+^ B cells and of the follicular (CD21^+^CD23^+^) and marginal zone (CD21^+^CD23^−^) B cells in the spleen of *Vav*-Cre*P2*^fl/fl^ mice revealed that B cell development was comparable to that in WT mice (Fig. 7e and Supplementary Figure S3b). These observations are in stark contrast to the arrested B cell development in *Vav*-Cre*Nfatc1*^fl/fl^ mice, which led us to investigate whether NFATc1 activity was still present in pro-B cells in *Vav*-Cre*Nfatc1P2*^fl/fl^ mice. RT-PCR analysis revealed a complete loss of P2 promoter activity, but surprisingly, in the absence of *Nfatc1b*, robust expression of *Nfatc1a* directed from the P1 promoter was observed in *Vav*-Cre*Nfatc1P2*^fl/fl^ mice (Fig. 7f). Thus the normal B cell development in *Vav*-Cre*Nfatc1P2*^fl/fl^ mice was clearly due to compensation for the lost NFATc1β activity by the NFATc1α isoforms. These observations further underline the importance of NFATc1 in B cell development.

To gain insight concerning the absence of NFATc1α expression at the pro-B cell stage, when it is present in pre-B cells along with NFATc1β (Fig. 7a) and is able to replace NFATc1β activity during early B cell development (Fig. 7f), we analyzed *Vav*-Cre*Nfatc1*αA^fl/fl^ mice in which a constitutively active version of *Nfatc1*α*A* was knocked-in in the *Rosa-26* locus flanked by a floxed stop cassette.^34^ Surprisingly, analysis of the spleen and BM revealed an NFATc1αA dose-dependent decrease in cellularity, suggesting a possible defect in B cell development in *Vav*-Cre*Nfatc1*αA^fl/fl^ mice (Fig. 7g). Characterization of the B cell populations revealed a drastic reduction of B220^+^ cells in the BM and spleen (Fig. 7h and Supplementary Figure S4a). Furthermore, we observed a severe lack of IgM^+^ and IgD^+^ B cells in the BM and spleen, as well as follicular and marginal zone B cells in the spleen of *Vav*-Cre*Nfatc1*αA^fl/fl^ mice compared with their littermate controls (Fig. 7i, j and Supplementary Figure S4b and c). Additionally, the development of B220^+^CD19^+^CD5^+^ B cells in the spleen was also severely affected in *Vav*-Cre*Nfatc1*αA^fl/fl^ mice (Fig. 7k and Supplementary Figure S4d). To locate the precise stage at which B cell development was arrested as a result of NFATc1αA co-expression along with NFATc1β, we analyzed the distribution of B220^+^CD19^+^IgM^−^CD25^+^ pre-B cells in the BM of *Vav*-Cre*Nfatc1*αA^fl/fl^ mice. A drastically reduced pre-B cell population in the BM revealed that, similar to the *Vav*-Cre*Nfatc1*^fl/fl^ mice, B cell development in *Vav*-Cre*Nfatc1*αA^fl/fl^ mice was blocked at the pro-B cell stage (Fig. 7l and Supplementary Figure S4e). These observations suggest that NFATc1α activity in addition to NFATc1β activity at the pro-B cell stage is detrimental for B cell development, and a certain threshold of NFATc1 activity irrespective of the α or β isoforms is indispensable for normal B cell differentiation.

## Discussion

The role of NFAT proteins in early B cell developmental stages, during which they do not even have a pBCR, is not known. Our findings concerning NFAT protein expression in pro-B cells are quite intriguing (Fig. 1a–d and Supplementary Figure S1a and b). A previous report investigating the role of NFATc2 and NFATc3 in B cell development did not observe any abnormality in the BM, although the function of mature B cells was affected.^36^ This phenomenon could be due to a functional compensation by NFATc1 in the absence of NFATc2 and NFATc3. *Nfatc1*^−/−^ mice are embryonic lethal.^48^ Therefore, in an attempt to define the role of NFATc1 in B cells, we have previously used conditional mutant mice in which NFATc1 activity is ablated in a B cell-specific manner using *Cd79a* (*Mb1*, *Iga*)-Cre transgenic mice (*Mb1-CreNfatc1*^fl/fl^).^37^ Although Igα is a component of the pBCR and is involved in pBCR signaling, analysis of *Mb1-CreNfatc1*^fl/fl^ mice did not reveal any defects in early B cell development.^37^ However, functional defects in mature B cells were observed. These findings suggested that the absence of NFATc1 activity at a stage when B cells have already acquired the pBCR might not be appropriate to unravel its role in pBCR-negative stages of differentiation. Accordingly, *Vav*-Cre-mediated ablation of NFATc1 activity demonstrated the critical role played by NFATc1 in pro-B cell differentiation, which was not compensated by NFATc2 or NFATc3 (Figs. 2 and 3). The severe B cell lymphopenia observed in the absence of NFATc1 activity (Fig. 2c, d) was not present in mice that were deficient in NFATc2, NFATc3, or both (Fig. 1e). These observations attribute a specific role to NFATc1, in contrast to the other NFAT family members, in regulating B cell development in the BM.

Applying multiple approaches, we demonstrated the expression of NFATc1 in pBCR-negative pro-B cells (Fig. 1a–d, f Supplementary Figure S1a and b, and Fig. 7a). The functional significance of NFATc1 in these cells was clearly demonstrated by the B cell lymphopenia observed in *Vav*-Cre*Nfatc1*^fl/fl^ mice (Fig. 2c, d). In contrast to pBCR- or BCR-bearing B cells, pBCR-negative pro-B cells employ the alternative IL-7-Jak3-mediated mode to activate NFATc1,^33^ as evidenced in our kinase assays with immunoprecipitated JAK3 from pro-B cells (Fig. 1j, k). The role of NFATc1 in B-1 B cell development has been previously reported.^35^ Our analysis of NFATc1 ablation at the earliest stages provides evidence that it is indispensable for the development of all types of B cells (Figs. 2 and 3).

The block at the pro-B cell stage in *Vav*-Cre*Nfatc1*^fl/fl^ mice (Fig. 3f, g) was similar to that previously reported in *Il7*^−/−^ and *Il7r*^−/−^ mice, suggesting that the interruption of IL-7-NFATc1 signaling might be the cause of this phenotype. EBF1 deficiency has been reported to be the main factor underlying the developmental arrest at the pro-B cell stage in *Il7*^−/−^ and *Il7r*^−/−^ mice.^47^ Regulation of *Ebf1* expression by IL-7 signaling has also been reported.^16^ However, the molecular mechanism linking IL-7 and *Ebf1* expression is far from clear. Our observations regarding a strong defect in *Ebf1* expression in pro-B cells from *Vav*-Cre*Nfatc1*^fl/fl^ mice (Fig. 4a) suggested that, similar to *Il7*^−/−^ and *Il7r*^−/−^ mice, the loss of EBF1 activity could be the major factor responsible for the impaired B cell phenotype in these mice. Although we also observed downregulated *Pax5* expression (Fig. 4a), it is not likely to be the reason for the observed phenotype because EBF1 has been shown to regulate *Pax5* expression.^16^ Moreover, the strong downregulation in pBCR component expression (*Cd79a*, *Cd79b*, *Vpreb*, *Igll1*) and absence of *Rag1*, *Rag2*, and *Tdt* expression in *Vav*-Cre*Nfatc1*^fl/fl^ mice (Fig. 4a) resembled the phenotype of EBF1-deficient mice, in which B cell development is also arrested at the pro-B cell stage.^12^ Interestingly, we observed an increase in the expression of *Oct1*, *Oct2*, *Rela*, *Relb*, *Nfakb2*, and *Spi1* in *Vav*-Cre*Nfatc1*^fl/fl^ compared with littermate control mice (Fig. 4a); however, increased expression of these genes failed to rescue the B cell defects in *Vav*-Cre*Nfatc1*^fl/fl^ mice. Additionally, the higher levels of *Tcf12*, *Tcf3*, and *Id* protein (*Id1*, *Id2*, and *Id3*) expression in *Vav*-Cre*Nfatc1*^fl/fl^ pro-B cells had no beneficial influence on B cell development (Fig. 4a). Overall, the gene expression defects in *Vav*-Cre*Nfatc1*^fl/fl^ pro-B cells were similar to those observed in IL-7 signaling-deficient mice (Supplementary Figure S2b). Most interestingly, despite the block at the pro-B stage and the elevated expression levels of Notch proteins (Fig. 4a), no increases in T lineage cells were observed in *Vav*-Cre*Nfatc1*^fl/fl^ mice. Our analysis clearly demonstrates that the dysregulated *Ebf1* expression in *Vav*-Cre*Nfatc1*^fl/fl^ pro-B cells is the result of a lack of NFATc1 activity (Fig. 5). The defect in V-D-J recombination in *Vav*-Cre*Nfatc1*^fl/fl^ pro-B cells (Fig. 6) again was similar to that reported in *Il7*^−/−^, *Il7r*^−/−^, and *Ebf1*^−/−^ mice.^16,55^ IL-7 signaling and EBF1 have been shown to regulate Ig gene rearrangement.^16,52,53^ Again, the similarity in this aspect suggests that IL-7, NFATc1, and EBF1 all act in a linear axis (Supplementary Figure S5a) and that a deficiency in any one will lead to similar defects in B cell development.

In pro-B cells, *Nfatc1* expression occurred exclusively from the P2 promoter, whereas in pre-B cells we could detect *Nfatc1* expression from both the P1 and P2 promoters (Fig. 7a), marking a qualitative and quantitative difference in NFATc1 proteins in pro- and pre-B cells. This finding is again similar to our results obtained for pTCR-negative and pTCR-positive thymocytes during T cell development.^33^ In addition, this differential promoter-directed NFATc1 expression in pro- and pre-B cells reflects the threshold of NFATc1 activity required for their differentiation, as evidenced in our analysis of *Vav*-Cre*Nfatc1P2*^fl/fl^ mice, where in the absence of P2, P1 promoter activity compensated for the loss of NFATc1β and facilitated normal B cell development (Fig. 7d, e and Supplementary Figure S3). Additionally and stressing the importance of a threshold level of NFATc1 activity during a stage-specific differentiation of B cells, increased NFATc1 levels via the co-expression of NFATc1α along with NFATc1β in pro-B cells led to severe B cell lymphopenia (Fig. 7g–l and Supplementary Figure S4).

In summary, our study underlines the essentiality of an optimal level of NFATc1 activity for B cell development and for preventing B cell lymphopenia (Supplementary Figure S5b). Our study also raises the possibility of manipulating NFATc1 activity to provide relief in clinical cases of B cell immunodeficiency.

## Electronic supplementary material

Supplementary Information
