# Supplementary material for: B cell development is critically dependent on NFATc1 activity
Source: Cell Mol Immunol. 2018 Jun 15;16(5):508–20. doi: 10.1038/s41423-018-0052-9 (PMC6474210; doi:10.1038/s41423-018-0052-9)

Supplementary Information

B cell development is critically dependent on NFATc1 activity

Sabrina Giampaolo[1], Gabriela Wójcik[2], Stefan Klein-Hessling[1, 3], Edgar Serfling[1, 3] & Amiya K. Patra[1, 2]

SUPPLEMENTARY INFORMATION

SUPPLEMENTARY FIGURE LEGENDS

Supplementary Figure S1. NFATc1 expression at different stages of B cell development. (**a**) Immunofluorescence analysis of NFATc1, NFATc2 and NFATc3 expression in B220$^+$IL-7R$^+$ pro-B and B220$^+$IL-7R$^-$ pre-B cells from WT mice. (**b**) Quantification of nuclear NFATc1 (pro-B; *n* = 21 and pre-B; *n* = 34), NFATc2 (pro-B; *n* = 14 and pre-B; *n* = 34) and NFATc3 (pro-B; *n* = 17 and pre-B; *n* = 35) levels in WT pro- and pre-B cells. (**c**) Mean fluorescence intensity (MFI) of GFP levels in B220$^+$CD19$^+$IgM$^-$c-Kit$^+$ pro-B, B220$^+$CD19$^+$IgM$^-$CD25$^+$ pre-B, B220$^+$CD19$^+$IgM$^+$IgD$^-$ immature B and B220$^+$CD19$^+$IgM$^+$IgD$^+$ mature B cells in the bone marrow from *Nfatc1-eGfp-Bac* tg mice. (**d**) GFP expression representing NFATc1 levels in B220$^+$CD19$^+$IgM$^+$IgD$^-$, B220$^+$CD19$^+$IgM$^+$IgD$^+$ and B220$^+$CD19$^+$IgM$^-$IgD$^+$ splenic B cells from WT and *Nfatc1-eGfp-Bac* tg mice. Numbers inside each histogram represent MFI. Data are representative of three independent experiments and are shown as mean ± s.d., unpaired *t*-test.

Supplementary Figure S2. Defective B cell development and B cell-specific gene expression in absence of IL-7 signaling. (a) B220$^+$CD43$^+$CD24$^-$BP1$^-$ pre-pro-B, B220$^+$CD43$^+$CD24$^+$BP1$^-$ pro-B and B220$^+$CD43$^+$CD24$^+$BP1$^+$ large pre-B cells distribution in the BM from *Il7r*$^{-/-}$ *and Il7*$^{-/-}$ mice compared to WT controls. **(b)** RT-PCR analysis revealing expression levels of B cell development-specific transcription factors and pre-BCR-associated molecules in pro-B cells from *Il7*$^{-/-}$ mice compared to WT mice. Data represent one of three independent experiments (*n* = 4 per group per experiment).

Supplementary Figure S3. Normal development of B cells in *Vav*-Cre*Nfatc1P2*$^{fl/fl}$ mice. (**a**) Quantification of percent B220$^+$ cells distribution and of total B220$^+$ B cells in the BM and spleen of *Vav*-Cre*Nfatc1P2*$^{fl/fl}$ mice compared to WT littermates. (**b**) Quantification of percent

distribution and total cell numbers of IgM^+, IgD^+, IgM^+IgD^+, $CD21^+CD23^-$ and $CD21^+CD23^+$ cells in the spleen of *Vav*-Cre*Nfatc1P2*$^{fl/fl}$ mice compared to WT littermates. Data represent one of three independent experiments (*n* = 3 per group), and are shown as mean ± s.d., paired *t*-test, ns = not significant.

Supplementary Figure S4. Defective B cell development in *Vav*-Cre*Nfatc1αA*$^{fl/fl}$ mice. (**a**) Quantification of percent distribution and total cell numbers of $B220^+$ B cells in the BM and spleen of *Vav*-Cre*Nfatc1αA*$^{fl/fl}$ mice compared to WT littermates. (**b**) Quantification of percent distribution and total cell numbers of IgM^+, IgD^+ and IgM^+IgD^+ cells in the BM and spleen of *Vav*-Cre*Nfatc1αA*$^{fl/fl}$ mice compared to WT littermates. (**c**) Quantification of percent distribution and total cell numbers of $CD21^+CD23^-$ and $CD21^+CD23^+$ cells in the spleen of indicated mice. (**d**) Quantification of percent distribution and total cell numbers of splenic IgM^-CD5^+ cells in the spleen of *Vav*-Cre*Nfatc1αA*$^{fl/fl}$ mice compared to WT littermates. (**e**) Quantification of percent distribution and total cell numbers of $B220^+CD19^+IgM^-CD25^+$ pre-B cells in the BM of indicated mice. Data represent one of three independent experiments (*n* = 3 WT and 4 *Vav*-Cre*Nfatc1αA*$^{fl/fl}$ mice), and are shown as mean ± s.d., unpaired *t*-test.

Supplementary Figure S5. NFATc1 activity is critical for B cell development in bone marrow. (**a**) Schematic diagram showing the involvement of NFATc1 upon IL-7 signaling in regulating EBF1 expression. EBF1 in turn regulates Pax5 expression essential for B cell differentiation and lineage maintenance. EBF1, Pax5 and E2A activity is critical for the differentiation of pro-B cells to pre-B cell and later stages. (**b**) Model showing the essentiality of a threshold level of NFATc1 activity in B cell development. Either deficiency or supra-physiological NFATc1 activity is detrimental for BM B cell differentiation and will invariably lead to the development of severe B cell lymphopenia.

Supplementary Table 1

LIST OF RT-PCR and ChIP PRIMERS

Gene	Primer Sequence	Product Size
Actb	For: 5'- CCAGGTCATCACTATTGGCAAGGA -3'<br>Rev: 5'- GAGCAGTAATCTCCTTCTGCATCC -3'	223 bp
Calcineurin	For: 5'- AAGGAGGGAAGGCTGGAAGA -3'<br>Rev: 5'- GGCATCCATACAGGCATCAT -3'	608 bp
Cd19	For: 5'- CTCCTCTCCCTGTCTCCTTC -3'<br>Rev: 5'- CCACTATCCTCCACGTTCAC -3'	350 bp
Cd79a	For: 5'- AACCACAGGGGCTTGTACTG -3'<br>Rev: 5'- CCCCTGGAGATGTCCTCATA -3'	318 bp
Cd79b	For: 5'- TCCAGACCCTCCTCATCATC -3'<br>Rev: 5'- TATCCAGGAAGCAGGGAATG -3'	284 bp
Dntt	For: 5'- CTCCCAGAATGTTCCAGC -3'<br>Rev: 5'- ATGGGGAATGGCAGAGAT -3'	204 bp
Ebf1	For: 5'- TGCGGAAATCCAACTTCTTC -3'<br>Rev: 5'- GGTTCTTGTCTTGGCCTTCA -3'	257 bp
Ebf1a	For: 5'- AGAGGGCCTTTGAGCTTAGG -3'<br>Rev: 5'- AAAAGAGGGGATGGCTTTTG -3'	240 bp
Ebf1b	For: 5'- CCCTCCTTGTTATCGCTCAG -3'<br>Rev: 5'- CCTGGATCCCAAACATGAAA -3'	221 bp
Foxo1	For: 5'- CTGCCCAGTCTGTCTGAAAT -3'<br>Rev: 5'- GGGGAGGAGAGTCAGAAGTC -3'	345 bp
Id1	For: 5'- CCAGTGGGTCTCATCCCTTA -3'<br>Rev: 5'- AGAAATCCGAGAAGCACGAA -3'	368 bp
Id2	For: 5'- ACTCGCATCCCACTATCGTC -3'<br>Rev: 5'- TCCCCATGGTGGGAATAGTA -3'	453 bp
Id3	For: 5'- CTTGGCGGTCTGTTTTGAAT -3'<br>Rev: 5'- GTCAGTGGCAAAAGCTCCTC -3'	494 bp
Igll1 ($\lambda 5$)	For: 5'- GAGCTTCAGTGGGAAGCAAC -3'<br>Rev: 5'- GGGTAGAATTCGCTCACCAA -3'	268 bp
Il7r	For: 5'- AAAGCATGATGTGGCCTACC -3'<br>Rev: 5'- CAGGAAACTTTCGGGATTGA -3'	427 bp
Jak3	For: 5'- ACTGGCGGGACTTATCACAC -3'<br>Rev: 5'- GAAGTCCCTCTGCTGGTCTG -3'	352 bp
Nfkb1	For: 5'- GTGGAGGCATGTTCGGTAGT -3'<br>Rev: 5'- CCAAGTGCAGAGGTGTCTGA -3'	609 bp
Nfatc1	For: 5'-GACTTCGATTTCCTCTTCGAGTTC-3'<br>Rev: 5'-CTCGATTCTCGGACTCTCCAG-3'	297 bp
Nfatc1α:<br>(P1 activity)	For: 5'-GGGAGCGGAGAAACTTTGC-3'<br>Rev: 5'-GATCTCGATTCTCGGACTCTCC-3'	319 bp
Nfatc1β:<br>(P2 activity)	For: 5'-CGACTTCGATTTCCTCTTCGAG -3'<br>Rev: 5'- GATCTCGATTCTCGGACTCTCC-3'	311 bp
Nfatc2	For: 5'-GGGTTCGGTGAGTGACAGTT-3'<br>Rev: 5'-CTCCTTGGCTGTTTGGGATA-3'	371 bp
Nfatc3	For: 5'-CCGATGACTACTGCAAACTGTGG-3'<br>Rev: 5'-TTTGAATACTTGGGCACTCAAAGG-3'	343 bp

Notch1	For: 5'- TTGACGTCACTCTCCTGTGC -3'<br>Rev: 5'- ACACAGGTGCCATTGTTGAA -3'	408 bp
Notch2	For: 5'- ACCCTTGTATGCACGGAGTC -3'<br>Rev: 5'- CCAGGTTATTGCACGTTCCT -3'	373 bp
Notch3	For: 5'- GCACCTGCAACCCTGTTTAT -3'<br>Rev: 5'- TCTCCAGCATCACCACAGAG -3'	370 bp
Oct1	For: 5'- TTCAGTGCAGTCAGCCATTC -3'<br>Rev: 5'- GGCTTTGCTGAGGTAGTTGC -3'	425 bp
Oct2	For: 5'- GGAGCTGGAACAGTTTGCTC -3'<br>Rev: 5'- GATGCTGGTCCTCTTCTTGC -3'	310 bp
Pax5	For: 5'- GGGCTCCTCATACTCCATCA -3'<br>Rev: 5'- CGTCAAGTTGGCTTTCATGT -3'	321 bp
Plcg2	For: 5'- CAAGAGAGCGCTGGAGTTAG -3'<br>Rev: 5'- TCAAACCAGAGAGCCACTTC -3'	327 bp
Rag1	For: 5'- ACCATGTGTCAAGCCACAAA -3'<br>Rev: 5'- TGGCTACAGCTGAGGAAGGT -3'	332 bp
Rag2	For: 5'- TCTCTAAAGATTCCTGCTACCTC -3'<br>Rev: 5'- TGGAATTCACTGCTGGGGTAC -3'	563 bp
RelA	For: 5'- GACCATGGACGATCTGTTTC -3'<br>Rev: 5'- CGTGAAAGGGGTTATTGTTG -3'	431 bp
RelB	For: 5'- GACAAGGTGCAAAAAGAGGA -3'<br>Rev: 5'- GATTCCATGTGGATCAGAGC -3'	315 bp
Spi1	For: 5'- CGGATGACTTGGTTACTTACG -3'<br>Rev: 5'- GTAGGAAACCTGGTGACTGAG -3'	292 bp
Stat5a	For: 5'- ACTGTTCAACATCAGCAGCA -3'<br>Rev: 5'- TTCAGATTCCAGAGGTTTCG -3'	327 bp
Stat5b	For: 5'- ACTGTTCAACATCAGCAGCA -3'<br>Rev: 5'- AGGCATCAGATTCCAAAACA -3'	328 bp
Tcf12	For: 5'- ATTTATTCCCCTGACCACAC -3'<br>Rev: 5'- GTAGCACATGGATAGCATCA -3'	281 bp
Tcf3	For: 5'- GATCTACTCCCCGGATCACT -3'<br>Rev: 5'- GGCATGGTTATGCAAAAGAC -3'	375 bp
Vpreb	For: 5'- GAACCTGGGGTATCTGAGCA -3'<br>Rev: 5'- CTCATAGCAACACCGCAGAA -3'	296 bp
	**ChIP Primers**	
Ebf1	For: 5'- AGGAAAGAAACATCTTTGGTT -3'<br>Rev: 5'- TTTGTAGTAATCACAGGCCG -3'	235 bp

Supplementary Figure S1

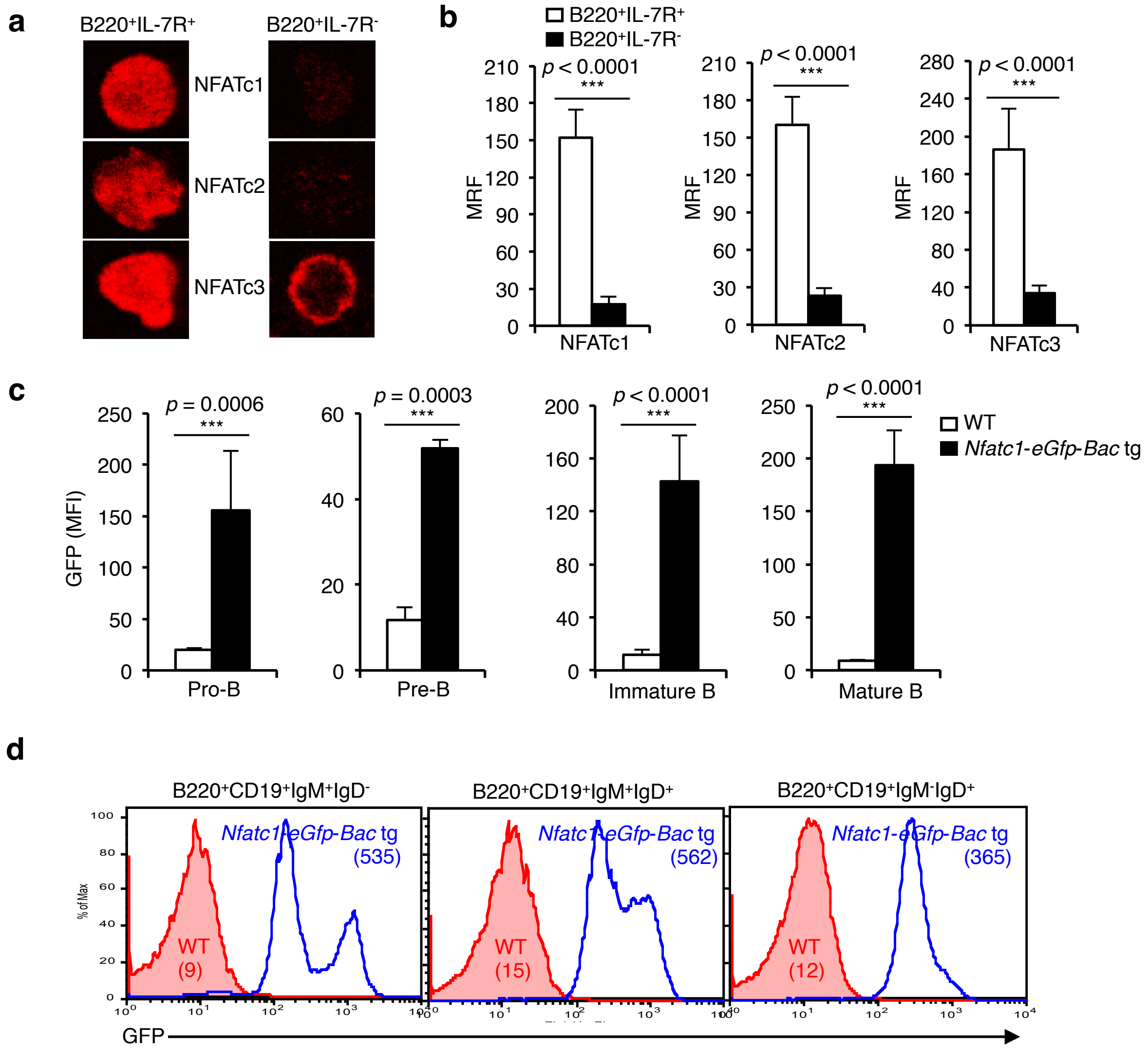

Supplementary Figure S2

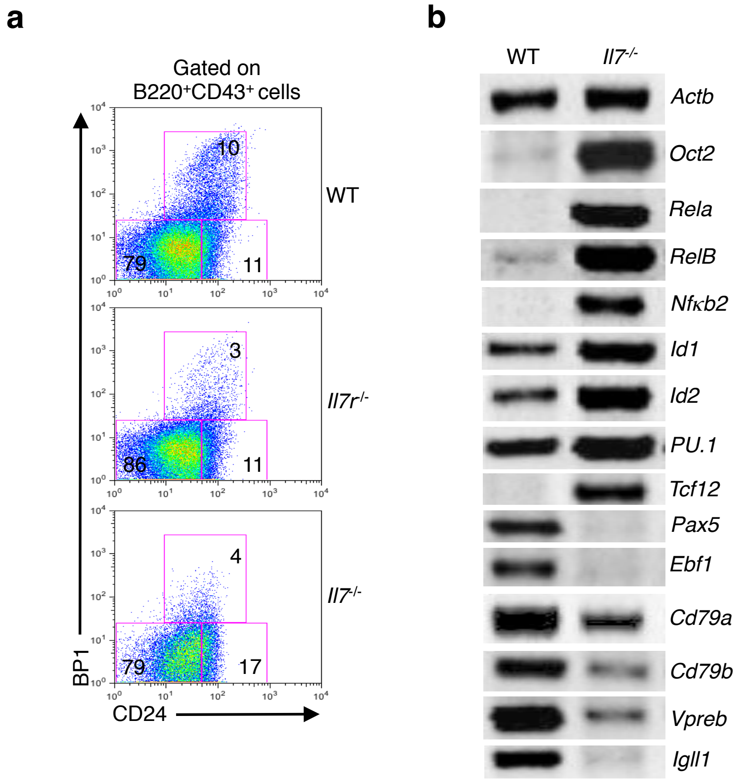

Supplementary Figure S3

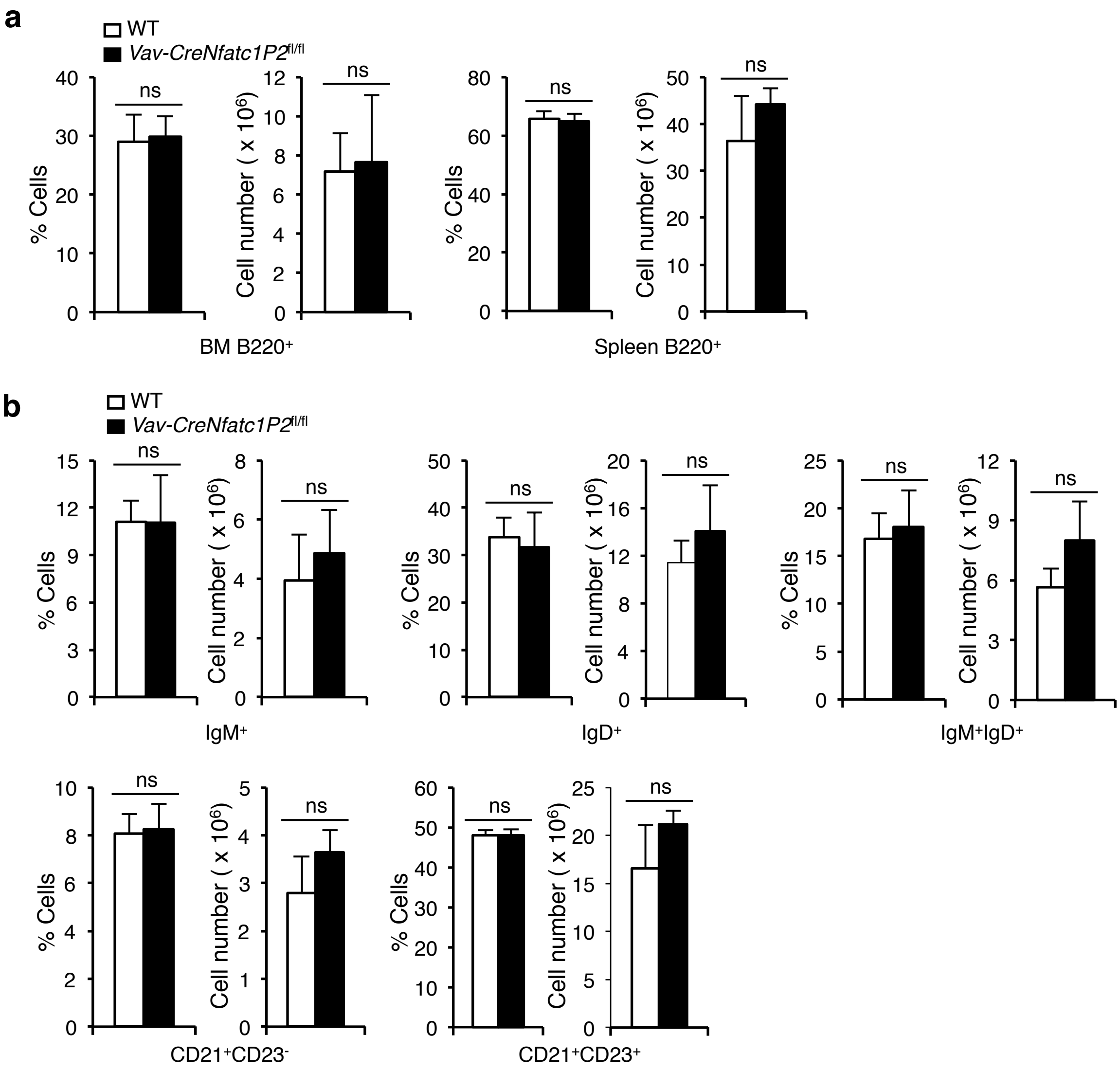

Supplementary Figure S4

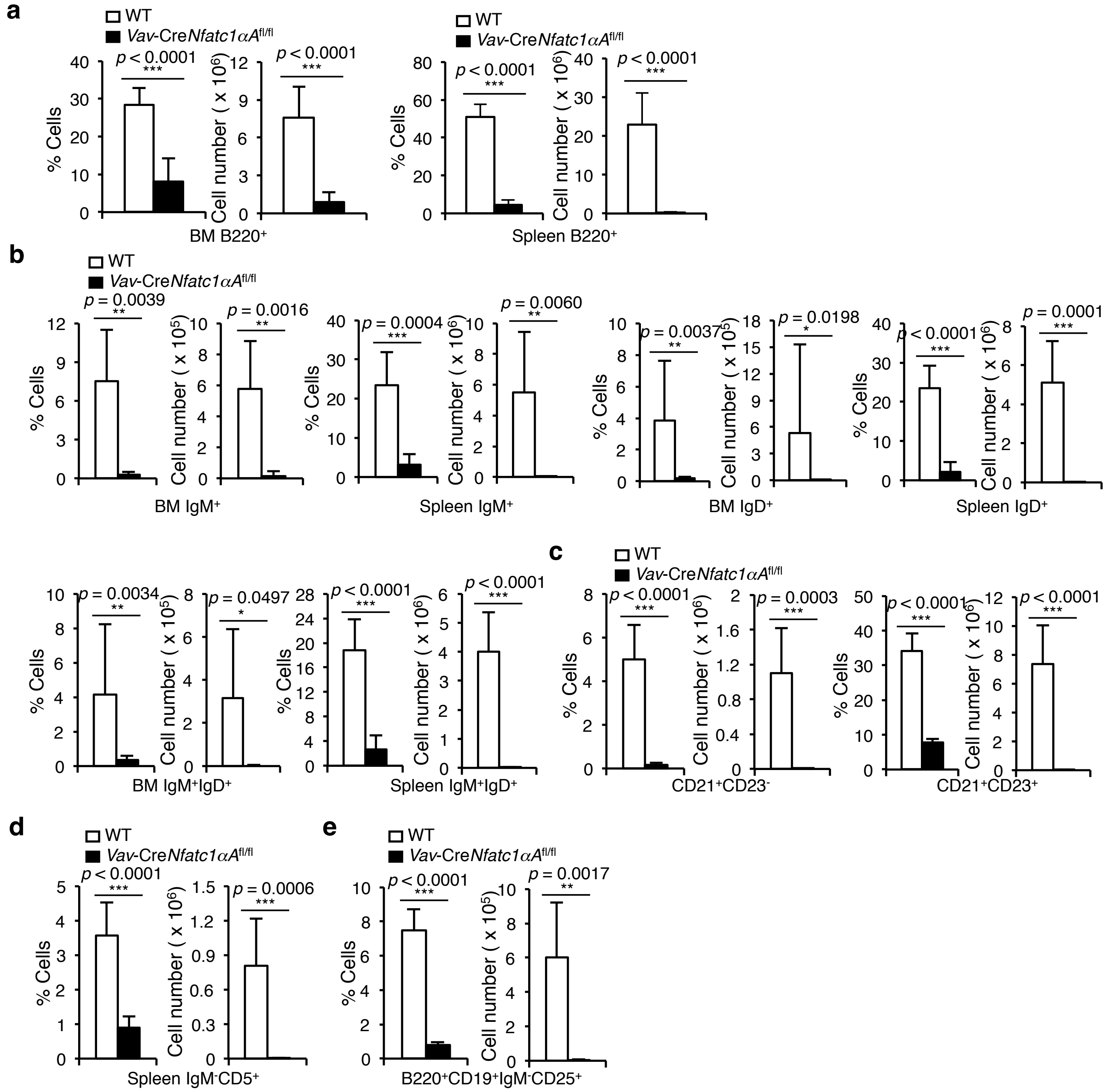

Supplementary Figure S5

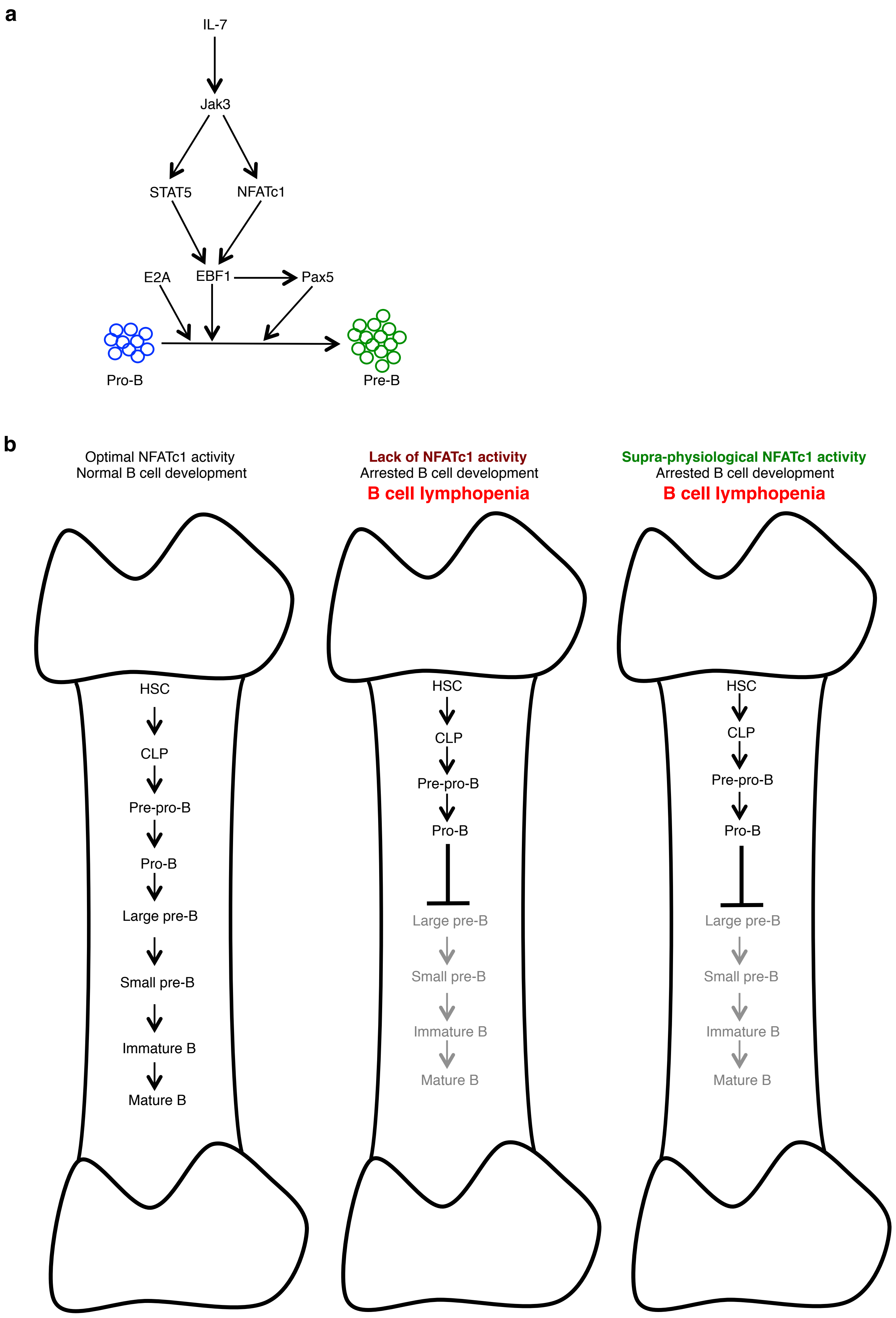

Supplement: Supplementary file 1 — Supplementary Information [file 41423_2018_52_MOESM1_ESM.pdf]
